# The identity construction of Iranian English students learning translated L1 and L2 short stories: Aspiration for language investment or consumption?

**DOI:** 10.3389/fpsyg.2022.972101

**Published:** 2022-11-04

**Authors:** Farangis Shahidzade, Golnar Mazdayasna

**Affiliations:** Department of English Language and Literature, Yazd University, Yazd, Iran

**Keywords:** identity reconstruction, imagined community, language consumption, language investment, learner identity

## Abstract

A large number of investigations have highlighted the importance of incorporating literary texts into English language teaching programs. Nevertheless, there are scarce studies on how short stories from L1 and L2 literature play a role in reconstructing learner identity in tertiary contexts. The present research study examines the identities of four non-native undergraduate students concerning aspirations for language investment or consumption. Data collection instruments were semi-structured interviews, open-ended questionnaires, and diary writings. The materials taught in the course consisted of three translated Persian and four target fictions related to loyalty and justice. The researchers used qualitative content analysis to explore the language learners’ self-images of initial, story-based, and prospective learning stages. Nearly all the participants regarded language learning as language consumption for pleasure in different stages except for the future aspiration stage; they did not aspire for personal economic benefits. The findings may enlighten curriculum designers in EFL contexts to incorporate literary texts from the native and target cultures into language education materials to provide opportunities for learners to experience diverse identities and meet individual preferences.

## Introduction

Many scholars have recently highlighted the role of literary texts in displaying and transmitting specific codes of the target culture (Komorowska, 2006; Fialho, 2012; Zunshine, 2015). Short stories can notably draw learners’ attention while dealing with themes from human experience (Hişmanoğlu, 2005). Therefore, cultural instruction impacts not only the cultural awareness of learners but also the reconstruction of self-identification (Norton and Toohey, 2011).

Exploring identity as the interrelationship between language learners and their socio-cultural contexts has drawn much attention in the current decades (Norton, 2000, 2013; Norton and Toohey, 2011). Most investigations on identity framing of language learners were conducted in second language contexts (Norton, 2000, 2013; Li and Seedhouse, 2010).

Despite the interwoven relationship between language learning, learner identity, and literature (Hişmanoğlu, 2005; Schrijvers et al., 2016), few studies have investigated how short stories from native and target cultures impact identity reconstruction in foreign language contexts. The previous investigations have already focused on the impacts of teaching original English literary texts on L2 learners’ identity (Li and Seedhouse, 2010; Shin and Riazantseva, 2015; Rezaei and Naghibian, 2018).

Highlighting the shortcomings of the literature, we can understand that there is a paucity of studies addressing how Iranian English learner identity is associated with learning short stories from both Persian and English cultures integrated into language teaching materials. This aspect of identity reconstruction, nevertheless, has remained under-researched. Accordingly, understanding the impacts of short stories of the Iranian readers ‘source and target cultures on their self-perceptions remains limited. It is indispensable to provide detailed information about incorporating stories into language teaching programs and their effects on language learners’ self-identifications.

This study attempted to explore language learners’ identity construction in the EFL classrooms through the new lens of aspirations to consume language for pleasure or to invest in language for capital return. It would investigate how their identity construction is associated with learning short stories from both native and target cultures. Accordingly, the study was conducted to address the following questions:

1. How are the Iranian English students’ identities represented in their initial experiences of learning the target language, English?
2. How are the Iranian English students’ identities constructed in the instructional stages of learning language through both the Persian stories translated to English and the target ones?
3. How are the Iranian English students’ identities represented in their future identities?

In the following section, we present some justifications for incorporating literary texts into language teaching programs. Next, the theoretical and practical frameworks pertinent to the learners’ identity reconstruction are clarified. Then, we explain the relationship between learner identity, language investment, language consumption, and imagined communities.

## Literature review

Over the past two decades, there is a growing trend to explore the effects of incorporating target culture literature on L2 learners’ identity (Hişmanoğlu, 2005; Yuen, 2011; Chinh, 2013; Shin and Riazantseva, 2015; Rezaei and Naghibian, 2018). Rezaei and Naghibian (2018) taught 12 American short stories to thirteen Iranian English learners to investigate the role of American short stories in reconstructing their cultural identity. Rezaei and Naghibian (2018) reported the participants’ respect for their source and target cultures. The findings presented the emergence of some patterns of change, such as being more active agents in the classroom discussions and thinking more critically while reconstructing meanings. Grounded in contemporary identity theories, Shin and Riazantseva (2015) conducted a case study on the identity construction of three Korean students engaging with an L2 novel. They explored how the EFL readers reconstructed their identity while constructing the meaning of an English novel named *The Catcher in the Rye.* The data collection instruments were semi-structured interviews, think-aloud protocols, and participants’ written responses. The thematic cross-case analysis showed that L2 readers’ engagement in reading the literary work contributed to emerging multiple identities at personal and social levels. According to the findings, multiple identities emerged while the readers encountered contrasting concepts of different cultures and contexts.

In contrast with the present study highlighting teaching materials from the source culture, Shin and Riazantseva (2015) incorporated the literary text only from the target culture. Few investigations have focused on how incorporating literary texts, in the target language but from the learners’ culture, into language teaching can impact language learner self-images. Schrijvers et al. (2016) employed diaries to explore whether the representative samples of Dutch students reading literary texts of originally Dutch culture reported some learning experiences affecting their self and social perceptions. The results presented that the experience enhanced readers’ potentials to modify their perceptions, imagine the situation of others, and empathize or sympathize with them. The study was, thus, limited to investigating originally Dutch texts. Similar to the current study, the researchers applied learner reports to investigate their self and social perceptions.

Considering the previous studies, however, it is unclear how incorporating short stories from both L1 and L2 literature, as practical teaching artifacts, plays a role in reconstructing L2 learner identity in the context of tertiary education. The present study intends to explore the relationship between learners’ past, current, and ideal L2 self-images and language learning through story-based instructions incorporated into the English teaching program. The impetus for conducting the investigation stems from the need to accurately design a comprehensive framework to analyze learners’ identity in terms of their past and present language-related experiences and their ideal expectations and aspirations of learning the language. The new model must effectively complement the identity construction theories of Norton (2013) as they undermine L2 learners’ aspirations to invest in learning L2 for capital return and do not consider aspirations for affective purposes. Therefore, the lack of a comprehensive analytical framework that facilitates exploring the learners’ identity concerning their desires for language consumption or language investment provided the impetus for this research.

## Theoretical and practical frameworks

The researchers employed a hybrid process of deductive and inductive thematic analysis (Swain, 2018). First, within the social constructionist paradigm, they selected the identity frame presented by Coll and Falsafi (2010) and the one implemented by Ushioda and Dörnyei (2009). Coll and Falsafi (2010) conceptualized learner identity as the two modalities of situated/short timescale and the cross-situational/long-timescale constructions impacted by a large bulk of formal along with informal educational experiences in diverse contexts. They considered the instructional settings of formal education like classrooms as the micro-context and the socio-cultural contexts as the macro-context mediating learners’ meaning construction about themselves. Ushioda and Dörnyei (2009) analyzed learners’ identity through their past, present, and future self-images. Building on the two frameworks, the researchers deductively analyzed the data related to the participants’ situated experiences while engaging in instructional activities associated with short stories of native and target cultures. Concerning the cross-situational/long-timescale dimension of identity construction, we investigated their past experiences and future identities. Next, we applied inductive coding to identify themes based on the data derived in the story-based stages.

**FIGURE 1:**
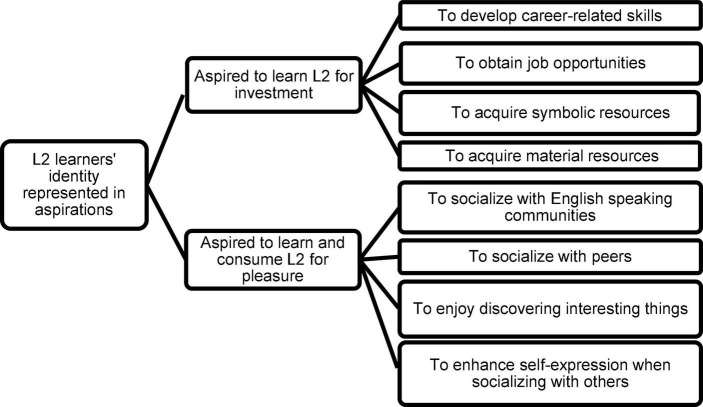
The new analytical framework of learner identity in terms of aspirations (based on Chen et al., 2020, p. 5).

Moreover, the researchers applied theoretical triangulation (Renz et al., 2018) to put new lenses into the frames analyzing language learners’ identity representations. We revised the operational model designed for learners of languages other than English (Chen et al., 2020), based on the analytical framework of Kubota (2011) concerning social inclusion in imagined communities, to deductively analyze the codes of the post-story stage. The new model highlights the two dimensions of identity represented as aspirations to invest in language learning or consume language for pleasure. The comprehensive model (Figure 1) redesigned and applied by the current researchers is as follows:

### Language learner identity and investment

According to Norton (2013), language investment contributes to understanding learners’ commitment to participate in social interactions and community practices. They tend to acquire a multitude of resources- the material types such as money or jobs, and the symbolic ones, including education and language, boosting their cultural capital and social power. As their capital increases, they restructure their current self-perceptions and their imagined identities to seek better futures. Similarly, Teng (2019) considered the conflict between the present community of practice and the imagined one as the fundamental factor affecting learner identity reconstruction. As Darvin and Norton (2017) noted, owing to the complexity and dynamicity of learners’ identity, their investment can be dynamic and complex leading to different learning outcomes. Other authors (Chen et al., 2020) have also considered language learners as potential investors with multiple context-congruent identities. Accordingly, investment and identity indicate the dynamic relationship between L2 learners and their willingness to practice and learn the language. Following the social constructionist approach, we employed the objectives explained by Norton (2013) to devise the section related to L2 learners’ aspirations for language investment (Figure 1).

### Language learner identity and language consumption

In contrast to the instrumental perspective of Norton (2000) regarding second language learning as an investment for gaining material and cultural resources, Kubota (2011) considered language learners’ engagement in language learning activities as a form of service consumption for enjoyment. In Kubota’s view, investigating the neglected dimension of language learning as the learners’ propensity to use language for pleasure in EFL contexts, where foreign language learners engage in language learning for various purposes, seems plausible.

Investigating the economic and personal dimensions of learning English in Japanese learners in informal settings through a qualitative study of male and female adult learners of English, Kubota (2011) reported the learners’ aspirations of socializing with local and global communities and joining those communities. Class observation, interviewing the participants and the language program providers were different sources of data collection. As Kubota (2011) noted, language learning is serious leisure since the systematic pursuit of accumulating and expressing some competencies provides learners’ self-fulfillment, self-actualization, and social inclusion in their imagined communities. The findings also revealed that many participants enjoyed inclusion for socializing with their peers and language teachers. They also longed to resemble other Japanese fluent in English. Kubota (2011) concluded that not only the hope for capital return but also the pleasure of language consumption is related to their desire for social inclusion into local or global communities. Considering the findings of the latter study, we devised the aspiration for pleasure section of the new analytical model to analyze the participants’ aspirations (Figure 1).

### Language learner identity and imagined communities

Imagined community as an underlying concept in second language learning identity refers to the aspiration community, where L2 learners are willing to belong. Language learners tend to be active members of communities of practice not directly accessible but empowering them against being the passive constituents of their socially imposed communities (Norton and Toohey, 2011). According to Ushioda and Dörnyei (2009), to reduce the gap between their current selves and aspiration and to realize their desire to be aligned with the communities, language learners tend to learn and use the target language. They acknowledged that investment illuminates the relationship between L2 motivation and language learner imagined identity.

Classifying imagined communities into national or transnational, Norton and Toohey (2011) highlighted their significant impacts on learners’ investment in second language learning. The classification was applied to analyze the data associated with the participants’ inclusion into expected communities (Table 6). After selecting and adopting the appropriate frameworks, the researchers employed the following methodology and design to answer the research questions.

## Materials and methods

Hiver and Al-Hoorie (2019) claimed that qualitative methods make provisions for in-depth data-driven explanations underlying the complex learning process. Moreover, as a complex system encompassing various components of diverse power and social relations, language classrooms required qualitative research. Noteworthy is that it is a part of a larger qualitative project on the impacts of teaching short stories on the identity construction of Iranian EFL learners. The study draws on the data for four learners from the larger dataset, highlighting their aspirations to invest in language learning or consume English.

### Design of the study

Before initiating the investigation, the researcher encountered the problem of selecting the most appropriate methodology. As Larsen–Freeman (2019) notes, the methodological options for exploring learner identity construction are not fixed and clear-cut due to the complex and dynamic nature of the construct, identity construction. The choice the researchers have made to make the exploration more manageable is data triangulation through several instruments. Accordingly, the researchers have conducted a qualitative case study using different data collection methods, including narrative interviews, questionnaires, and diary studies to investigate the relationship between English learners’ previous, current, and ideal L2 identities and their impacts on their investment in social interactions. Applying multiple sources of data collection has contributed to the rich description and interpretation of data. It has also provided the opportunity to validate the data.

Correspondingly, this design has been selected because case studies work as an effective way of investigating the why and how of current social events not controlled by the researcher in the actual context (Yin, 2012). Choosing the case study can also be attributed to its potential to function as the most practical means of investigating the attitudes, views, emotional status, and other factors associated with learner identity representation (Moore, 2016). Noteworthy is that in this case study, the intrinsic focus is not on generalization to other cases of identity construction, but the question raised is to know about the particular case of Iranian English students learning English through the native short stories and the target ones. The thematic data analysis by Saldaña (2016) has been applied to identify recurrent and common themes and categories among the data collected.

### Participants

The researchers arranged an initial online meeting with 35 English undergraduate students who were in the third semester and had enrolled for the compulsory course for BA students of English Literature entitled ‘Oral reproduction of short stories’ at Yazd University in the fall semester of 2021. They had already passed some courses in the first and second semester such as Reading Comprehension I and II (eight credits), Advanced Grammar I and II (eight credits), and Listening and Speaking I and II (eight credits). ‘Oral reproduction of short stories’ was the first course in which they were reading literary texts. The researchers introduced the requirements and benefits of this course in detail. The research project was not a part of the course program, and there was no course credit for the participation. Oxford Placement Test was used to assess the proficiency level of 25 students who were willing to participate in this research study. The test results revealed that fifteen were at upper-intermediate and advanced levels. The justification behind selecting the students of these levels is that they would not encounter serious language barriers to engage in classroom activities in an articulate manner. Accordingly, applying the standardized test of proficiency and selecting the participants of high proficiency would limit the effects of communicative language abilities on their language investment and identity reconstruction. The researchers provided the same interview environment for all participants to control the variable.

Among those fifteen students, the researchers selected four with maximum initial variations, including the way of initiating English learning and the years of learning English, intentionally. The team implemented purposeful sampling to analyze a range of diverse experiences. Investigating these varied information-rich cases (Patton, 2002) would help identify cross-case similarities and variations (Creswell, 2007). The participants were given pseudonyms for ethical issues. Regarding how they initiated language learning, Fatima and Arman had commenced it by attending language institutes, but for different purposes. While Fatima attended English classes to support her son as a helpful mother, Arman did it as a motivated school student. Whereas, Azin was motivated to study English by listening to English music, watching English films, and playing games in the beginning stages of learning English, Arshia’s condition was quite different due to living abroad and attending bilingual schools (Table 1). Concerning the length, Azin and Arshia recorded similar periods of sixteen years while the figures were dissimilar for Arman and Fatima, 7 and 5 years, respectively. They all were BA students at intermediate or upper-intermediate proficiency levels. Fatima and Azin were of upper-intermediate, but Arman and Arshia’s level was advanced. Table 1 presents the participants’ demographic data, including their age, gender, major, linguistic level, and the length and way of learning English.

**TABLE 1:** Participants’ demographic data.

Pseudonym	Age	Years of learning English	Gender	Major	Linguistic level	Way of learning English
Fatima	38	5	Female	BA/English Literature	Upper-intermediate	Attending L2 classes for adults in language institutes to help his son in English
Arman	21	7	Male	BA/English Literature	Advanced	Attending L2 classes for students in language institutes
Azin	22	16	Female	BA/English Literature	Upper-intermediate	L2 music, games and, films
Arshia	20	16	Male	BA/English	Advanced	Living abroad and attending bilingual schools

### Materials

The materials taught in the online oral presentation course consisted of three translated Persian and four target fictions^1^. The former included ‘*The Story of Shahrayar and Shahrazad*,’ *‘The Broken Cup,’ and ‘Justice’*; the latter were *‘A secret for Two,’ ‘Lamb to the Slaughter,’ ‘The Lady or the Tiger,’ and ‘One of These Days*.’ The stories incorporated loyalty and justice themes. They were of varied length, the highest of approximately 3,500 words and the lowest of less than 1,000 words, to be read and discussed in one or two sessions. The researchers selected them among several cultural themes because of their saliency in Persian culture. The native and target stories of loyalty and justice can represent cultural conceptualizations (Sharifian, 2017) of the themes within different cultural contexts. Reading the stories might provide opportunities for language learners to compare and contrast these conceptualizations and understand cross-cultural diversities. Regarding the Persian stories, the first two were of loyalty theme while the last was of justice. Concerning the target stories, the first two were of loyalty, but the rest were of justice.

During the semester, first loyalty and then justice stories were taught. Among the stories of each theme, the Persian and the target stories were taught, respectively.

The teacher (the second author) asked the students to read the story before attending each class session and be prepared to participate in class discussions. According to Mazdayasna (2012, p. 24) “the rationale behind this course is twofold: (1) to make students familiar with the literary elements used in short stories as regards characters and plot. It is assumed that through reading stories students can experience an enjoyable task, and come across universal themes, and foreign culture and values; (2) to reproduce the stories in the classroom in their own words to improve their speaking skills so that they become competent enough to speak appropriately and effectively.”

## Data collection and analysis

Investigating how learners’ identity construction and representation are linked to learning language through the source and target short stories required the researchers to have an intensive engagement with the participants for 9 months. To get informed consent from them, the researchers had a conversation with them about their willingness to participate in the study. The data collection process took over 8 months in a convivial atmosphere. The researchers applied different data collection methods to obtain a holistic and multi-faceted profile and avoid inaccuracies of one single source of data collection (Merriam and Tisdell, 2015). The data collection methods employed was as follows:

Studying the most relevant literature (Ushioda and Dörnyei, 2009; Norton, 2013; Sacklin, 2015), the researchers collected a wide range of statements and converted them into a set of open questions for the questionnaires and interviews. Three professors in applied linguistics reviewed the questions to see whether they could be appropriate for the given objectives. They offered some hints contributing to rewording and modifying them. Next, the researcher (first author) conducted a pilot study with three students of the same population to ensure the validity of confirmed questions. To initiate the first step of data collection, she sent the respondents open-ended questionnaires via email and they completed them.

The first researcher administered four questionnaires to give them space to record their personal and social experiences based on language teaching through the source and target short stories in their classes (Table 2). The first questionnaire gathered the participants’ demographic information about their gender, age, linguistic background, length of English learning experience, and educational status (Table 1). It also included some questions about their first experiences of EFL learning, self-images, and attitudes toward English native speakers and their culture. The second and third questionnaires collected some data about their present self-images and changes in their current interactions and connections to other cultures after involving in the story-based activities. The fourth questionnaire explored how their aspirations, related to language learning, were constructed over the period.

**TABLE 2:** Data collection through interviews, diaries, and questionnaires.

Data collection method	Data collection period	Data collected
		
	Fall semester of 2021	
Four open-ended questionnaires		
The first	The 1st session	Demographic information and initial experiences of learning English
The second	30 min during the semester	Self-perceptions after reading the native and target stories of loyalty, respectively
The third	30 min during the semester	Self-perceptions after reading the native and target stories of justice, respectively
The final	30 min during the last session	Future aspirations related to language learning non-intrusive diaries
Nine diaries		
Over the semester	Initial and present	English learning experiences and aspirations
Nine semi-structured interviews		
The first	45 min of the 2nd session	Videotaped narratives of past experiences
The next seven interviews	30 min after each story	Audio-taped emerging self-perceptions
The last	45 min of the final session	Audio-taped prospective aspirations

Following Norton (2013), we asked the participants to write nine diaries during the given semester (Table 2). We provided some guidelines for the participants to follow since they were not familiar with writing diaries. We asked them to recollect their initial memories of language learning and present learning experiences through stories throughout the investigation. They wrote them down in English or Persian every week during or after reading each story. Employing the framework of Ushioda and Dörnyei (2009), the researchers also asked them to record their plans and aspirations related to learning English in the last session.

Articulating their aspirations linked to their language learning was the next requirement. The researchers interviewed the participants through the items, such as asking them to compare their current self-image to the initial one of the beginning stages of learning English (Appendix A). In all WhatsApp interviews, they had an option to select their language since the focus was not on language but on reporting their individual experiences. To enhance the consistency of data, the investigators audio-taped and transcribed the responses. Recording the data might provide opportunities for the researchers to retrieve the data when required. It could also allow them to concentrate on the interviewing process without being concerned about missing some statements. Roughly fifty pages of data were accumulated to be analyzed. Table 2 illustrates the data collection procedures.

To analyze the data of the initial stages of EFL learning, the story-based instruction phases, and the future aspirations of English learning, we designed a composite file for each participant, encompassing the data of different methods discretely. Regarding the data of the initial stages of language learning and story-based stage, applying the thematic analysis by Saldaña (2016), the researchers identified the codes after comparing and contrasting the data obtained through these methods. Next, we formed common categories from similar initial codes. Finally, we derived the themes of attitudinal and emotional aspects of the participants’ self-descriptions for the first stage and the attitudinal, affective, and social ones for the story-based stages (Appendix B). Regarding the post-story stage data, the first researcher employed a theory-driven analysis (Swain, 2018), based on the newly designed model, to identify the themes.

Considering the qualitative analysis of the data, the researchers focused more on the iterative points the participants pointed to as the data collection developed to extract more precise and detailed responses illuminating the relationship between their identity, investment, classroom community, and possible communities of practice. The analysis sheds light on how the participants evaluate their self-reconstruction throughout the course and how their participation within the classroom context would determine their investment in the current classroom and prospective communities.

During the data collection and analysis, there were regular WhatsApp contacts between participants and the researchers. To perform Member checks (Lincoln and Guba, 1985), we asked the respondents to comment on the analyzed data and provide us with feedback about the verification of quotes, codes, and interpretations. It aimed to check the authenticity of data and make amendments if necessary. Moreover, a professor in applied linguistics reviewed the entire procedures, including data collection and analysis, to perform external auditing. To conduct researcher triangulation, both researchers coded and made themes from the same data independently to ensure inter-rater reliability (Merriam and Tisdell, 2015). A high rate of agreement, 86%, was documented. The subtle differences observed were clarified during the regular team meetings, and the codes were modified later (Saldaña, 2016). These processes contributed to establishing the trustworthiness of the research.

## Findings

This section presents the participants’ identity construction in different stages, including the initial stages of learning English, the story-based learning phases, and the post-story one of aspirations. Regarding the subjective nature of the qualitative study and the individual-related nature of identity construction as the research question, we applied the procedures of Norton (2013), the eminent scholar in L2 learner identity, to discuss each case discretely. Highlighting their particular quotes of self-images in different stages could present a clear profile of the informants objectively. Exemplifying the specific evidence could set out their self-perceptions clearly and comparatively.

### Participants’ self-descriptions of the initial stages of learning English

Table 3 illustrates how the participants reported their attitudes and emotional states while recollecting their self-perceptions of the stage (Appendix B). While the female participants (Fatima and Azin) were not satisfied with the new experience, the males (Arman and Arshia) enjoyed it (Table 3). The data recording their attitudes showed individual differences.

**TABLE 3:** Participants’ recollecting self-descriptions of the initial phases of learning English.

Name	Emotional state	Attitudes toward English speakers and culture
Fatima	Embarrassed about the strange world of superiors (Dia-1-Oct-22-2019)	Focusing on substantial cultural differences (Int-1-Oct-22-2019)
Arman	Enjoying learning English (Ques-1-Oct-8-2019)	Considering western people attractive and their culture learnable (Ques-1-Oct-8-2019)
Azin	Not satisfied due to not having familiarity with L2 culture (Int-1-Oct-22-2019)	Considering L2 speakers as model teachers (Dia-1-Oct-22-2019)
Arshia	Pleased about learning English in an English speaking atmosphere (Ques-1-Oct-8-2019)	Highlighting L2 speakers’ superiority (Int-1-Oct-22-2019)

### Participants’ self-images in the native story-based instruction stage

Common cross-case themes and within-case analyses of this stage were derived from the codes. The inductive data analysis illustrated that all highlighted the three common categories of identities, including affective, attitudinal, and social dimensions. It showed how these aspects were affected by interacting with originally Persian stories (Appendix B).

Table 4 shows the common themes, including positive emotions, positive attitudes toward self-development, and active roles in class interactions that emerged across different participants in the stage.

**TABLE 4:** Emerging cross-case themes of the native story-based phase.

Positive emotions associated with Enjoying learning story-based Persian literature in English Admiring the stories offering a place for discussing current social issues Feeling closer to the original identity
Attitudes toward self-development Understanding the value of Persian literature and heritage Developing a positive attitude toward their cultural identity Consolidating the knowledge of Persian history, literature, and culture
Social interactions in the classroom A communicative learner with a large amount of ambiguity tolerance A story analyzer keen on sharing views from the Persian background

In the source story-based instruction phase, with-in case data are as follows:

#### Fatima’s case

She experienced varied feelings of excitement, admiration, and sadness while being engaged with Persian stories (Appendix B). She mentioned, “I like to learn the English equivalent forms of some concepts from Farsi through reading the stories to enhance my L2 proficiency level” (Int-6-Dec-10-2019). Concerning her attitude toward investment in interactions, she answered, “I prefer to discuss the stories in correspondence with my socio-cultural values. Similarity to our Islamic cultural values motivated me to participate in discussions” (Int-6-Dec-10-2019). Highlighting her social role as an active student, Fatima noted, “I only talk about the events in line with cultural values” (Dia-3-Nov-12-2019 and Dia-6-Dec-10-2019).

#### Arman’s case

Regarding his emotional status, he asserted, “As an expressive participant, I admire discussing the current social issues of the stories” (Ques-2-Dec-2019). Highlighting the value of Persian literature-based interactions, he mentioned, “Taking part in practices encourages me to discover, reconstruct, and express my values leading to the expansion of my identity” (Int-6-Dec-10-2019).

#### Azin’s case

To mention her attitudes toward class interactions, she noted that “Because of my personality, participation provides room for more interactions with the class members” (Dia-2- Nov-5-2019). She also responded, “I am an eager analyzer discussing the stories even when I am not positive” (Ques-3-Dec-31-2019).

#### Arshia’s case

Highlighting expressing characters’ rights in these classes, he mentioned:

It raises my confidence to express voice in other contexts, where social considerations do not let me defend my rights. Language learning in this context means self-confidence, self-expression, and self-development. … As a communicative learner, I appreciate Persian history and culture (Int-2-Nov-5-2019).

Concerning his attitudes toward learning Persian culture, not only did he attempt to strengthen his attachment to Persian identity, but he also developed his international identity by promoting the knowledge of English and its speakers’ culture. Analyzing the individual data indicates they tended to invest in Persian-story-based activities for varied purposes. Fatima preferred to discuss her cultural values while Arman participated in the stories of the current social issues. Azin, as an eager analyzer, desired to partake in all class activities, but Arshia participated for self-development.

### Participants’ self-images in the target story-based teaching stage

Categorizing the codes resulted in commonalities across the participants and within-case analyses. Like the previous stage, the data analysis of this stage revealed that all highlighted the affective, attitudinal, and social dimensions while describing self-images (Appendix B).

Table 5 shows the common themes of positive emotions of self-expression and intercultural awareness; positive attitudes related to boosting symbolic resources, self-image, and cultural flexibility; and social interactions enhanced by linguistic and cultural resources.

**TABLE 5:** Emerging cross-case themes of the target story-based phase.

Positive emotions associated with Discovering intercultural commonalities and contrasts Willingness to discuss intercultural commonalities and contrasts Having opportunities to express their personal views
Attitudes toward self-development Broadening L2 knowledge of symbols, words, and idioms Boosting self-image through discovering personal preferences Enhancing cultural flexibility
Social interactions in the classroom Increasing linguistic resources to enhance social interactions Discovering cultural commonalities and diversities through discussions

The participants highlighted their willingness to foster linguistic resources and discover commonalities and diversities between native and target cultures by participating in class discussions (Table 5). Their self-perceptions in this stage are as follows:

#### Fatima’s case

Fatima focused on her self-expressiveness when the first researcher asked about her willingness to participate in the story-based activities. She answered, “Participation promotes my self-image since I can express my emotions and views there” (Int-5-Nov-26-2019). She documented, “Nowadays, I adopt dissimilar perspectives more flexibly, contrasting my prior attitudes toward cultural differences. I feel more balanced in intercultural relations” (Ques-2-Dec-3-2019).

#### Arman’s case

Being asked about his attitudes toward learning English through target stories, he responded:

Sometimes I am reluctant to participate and learn English because of the complicated words and structures. I think I can strengthen my L1 and L2 cultural and linguistic repertoire through target stories. As an advocate of challenging and contrasting situations, I like to discuss English stories providing a chance for me to think and analyze issues deeply. However, I should consider sociocultural constraints in some settings (Int-8-Dec-24-2020).

#### Azin’s case

She deemed herself “a unique learner understanding the necessity of integrating short stories into language teaching to add pleasure” (Ques-3-Dec-31-2019). Concerning her purpose of class participation, she answered, “Experiencing the puzzling feelings of acceptance or rejection of cultural conceptualizations in target and source cultures motivates me. Clarifying the ambiguity and regulating emotions compel me to participate and learn English” (Int-4-Nov-26-2019).

#### Arshia’s case

Describing his self-reconstruction, he reported, “Challenging situations inspired me to vocalize my preferences. Offering opportunities to express myself made this experience special. To raise my self-confidence associated with my proficiency level, I always participated actively” (Dia-7-Dec-17-2020). Despite this, he mentioned some hindering factors affecting his willingness to participate, namely observing social structures imposing some constraints, confining him to discuss some cultural differences critically.

Concerning their different inspirations to participate in this stage, Fatima highlighted her self-expressiveness while Arman focused on his enthusiasm to encounter the challenges in the stories. Clarifying the ambiguity of different cultural conceptualizations motivated Azin, but Arshia desired to articulate his preferences and promote self-confidence.

### Participants’ aspirations in the post story-based stage

Following the perspective of Ushioda and Dörnyei (2009), the researchers explored L2 learners’ future selves through their aspirations to learn and use the language. Applying the deductive approach of managing data, we employed the new analytical model (Figure 1) to categorize learners’ aspirations into language investment or language consumption for pleasure (Appendix B).

Table 6 presents the common themes of language investment or consumption within imagined communities that emerged in this phase.

**TABLE 6:** Emerging themes of the participants’ future self-images.

Participants’ desire for language investment within imagined communities Expecting job opportunities in English-speaking communities Developing career-related skills in national communities Enhancing symbolic resources of source and target languages and cultures
Participants’ desire for language consumption within imagined communities To socialize with foreign tourists To communicate with foreigners for cultural awareness To raise self-expression and self-esteem

After learning language through target short stories, the data related to their future selves are as follows:

#### Fatima’s case

Concerning her desire for learning English, she writes:

Raising intercultural competence through studying foreign literature will make me a professional teacher. As a communicative teacher, I would like to communicate more with the tourists visiting my city. I can transmit my cultural knowledge to them and enhance my intercultural awareness in return. Learning about target culture helps me communicate with foreigners more efficiently, not only through face-to-face interactions but also the social media (Dia-9-Jan-21-2020).

#### Arman’s case

Regarding his aspirations related to language learning, he notes:

I would like to be a well-known writer writing in Persian. It enforces me to be a multilingual and multicultural writer. I am keen on studying and learning new languages and cultures other than English. In my view, this knowledge can lead to finding a new way to earn a living out of it. Besides, raising communication skills in the international language can increase my self-esteem (Ques-4-Jan-7-2020).

#### Azin’s case

Azin intends to be “a bilingual with rich linguistic and cultural competence who can communicate within English speaking communities well” (Ques-4-Jan-7-2020). She asserts, “To me, it means a lot to improve my proficiency. I love to convey the ignored cultural heritage of Persia to foreigners” (Int-9-Jan-21-2020). She expresses her willingness to be a multilingual literature professor at a foreign university, conveying Persian literature to foreigners.

#### Arshia’s case

While answering the question about his future interactions, he mentions:

I should make friendly relations with the target communities if I am going to be a successful tour guide. This course helped me read the literary texts of Iranian culture. It is a high preference for me. When my proficiency is high and cultural and intercultural knowledge is rich, I can make rapport easier and faster. While communicating with them, I can express my national dignity more effectively (Ques-4-Jan-7-2020).

To sum up their imagined careers, Fatima desires to become a professional English teacher, but Arman aspires to be a well-known writer in Persian. Azin’s imagined one is a multilingual literature professor, whereas Arshia’s is a successful tour guide.

### Participants’ self-perceptions in the pre, while, and post story-based stages

Table 7 summarizes how the participant described themselves in the initial stages of L2 learning, story-based phases, and post-story aspirations. It indicates how they reported their initial emotions and attitudes toward language learning, reconstructed their views while engaged with the stories, and portrayed their expectations related to language learning (Appendix B).

**TABLE 7:** Individual self-images in pre, while and post story-based instruction stages.

Participants	Initial stage perceptions	Persian story-based perceptions	Target story-based perceptions	Post-story aspirations
Fatima	Embarrassed about the strange world of superiors Focusing on substantial cultural differences	Experiencing varied feelings of excitement, admiration, and sadness while being engaged Discussing the stories in correspondence with sociocultural values learning the English equivalent forms of Farsi concepts to enhance L2 proficiency level for participation	Feeling more balanced in intercultural relations Positive emotions of expressing personal views Adopting diverse cultural perspectives more flexibly	Developing intercultural competence as a professional teacher Socializing with foreign tourists
Arman	Enjoying learning English Considering western people attractive and their culture learnable	Encouraged to expand identity through self-expression Discovering and reconstructing views	An advocate of contrasting issues but considering sociocultural constraints Strengthening L1 and L2 cultural and linguistic repertoire	Being a well-known multicultural Persian writer Increasing self-esteem through raising communication skills
Azin	Not satisfied due to not having familiarity with L2 culture Considering L2 speakers as model teachers	Being eager to analyze events even in ambiguity Interacting with the professor and classmates because of gregarious personality	A unique learner feeling the fun and pleasure of target stories Engaged to regulate the puzzling feelings of different conceptualizations in different cultures	A resourceful bilingual professor with rich linguistic and cultural competence at a foreign university Conveying Persian literature and culture to foreigners
Arshia	Pleased about learning English in an English speaking atmosphere Highlighting L2 speakers’ superiority	Feeling self-confidence, self-expression, and self-development. Appreciating Persian history and culture	Inspired to express personal preferences in challenges Raising self-confidence related to the proficiency level Experiencing hindering factors affecting his participation, namely social constraints	Making friendly relations with the target communities Being a successful multicultural tour guide Expressing national dignity Enriching proficiency level, cultural competence, and intercultural knowledge

To sum up, Table 7 reveals a multitude of intra-individual shifts within the stages of the investigation. For instance, Arshia’s and Fatima’s perceptions of inferiority compared to native speakers in their initial stages of learning English were not steady. Concerning the emotional dimension of identity reconstruction, the findings revealed remarkable alterations from anxiety and inferiority in the initial phases to positive self-perceptions and desires for membership in international communities (Table 7). While Fatima had been anxious about entering the world of superiors in the first stage, she adopted cultural differences more flexibly in the story-based stage, reaching the desire for socialization with foreign tourists in the last one. Commencing from highlighting the target community’s superiority, Arshia approached aspiration for friendly relationship with foreigners and becoming a multicultural tour guide in the post-story stage (Table 7).

The following sub-section illustrates the factors that emerged in the last stage, affecting the participants’ aspirations to consume or invest in English.

### Factors affecting language investment and consumption

**FIGURE 2:**
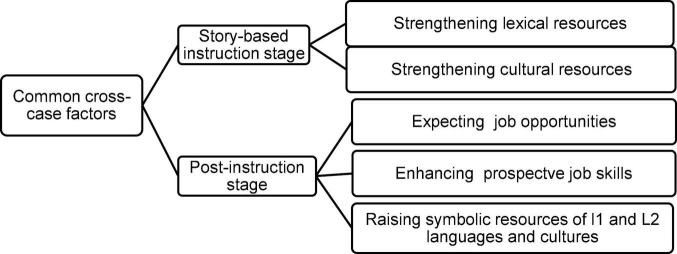
Common factors affecting language investment in the story-based phases.

**FIGURE 3:**
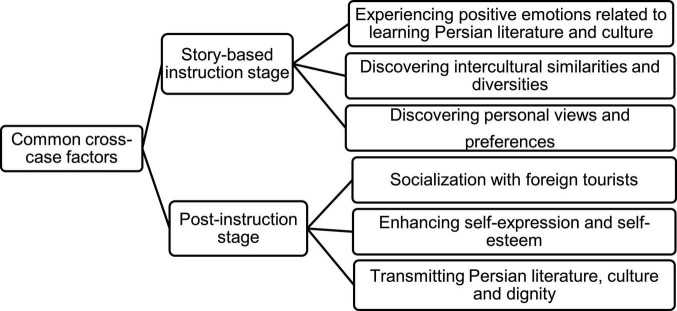
Common factors affecting language consumption in the story-based phases.

Analyzing the data has confirmed the perspectives of Norton (2013) about investment. As Figure 2 shows, the learners desire to invest in class practices to attain material resources, including job skills and opportunities, and symbolic resources-namely cultural and linguistic. All factors affecting investment found in this study are also present in the operational model (Figure 1). What is different is the participants’ aspirations to enhance L1 resources through reading short stories in the current findings. Accordingly, the model can be expanded as follows (see Figures 2, 3):

Confirming the results about L2 consumption for pleasure recorded by Kubota (2011), the findings present the common factors in the model (Figure 1) and in the current study (Figure 3). They include the EFL learners’ propensity for self-expression, socialization with foreigners, and discovering interesting things. Those specific to the given study are experiencing the positive emotions of learning and transmitting Persian culture, discovering intercultural and personal features, and socializing with foreign tourists, not the peers in the model (Figure 3).

## Discussion and conclusion

This study will likely broaden the understanding of English learners’ identity reconstruction and the relationship between identity construction and aspiration for investment in language learning and language consumption. The findings of different phases would provide adequate evidence for confirming the perspectives of Norton (2013) on the relationship between identity, investment, and SLA.

Applying the frameworks revealed that some facilitative and impeding elements in class communities impacted their potential language investment and consumption. In congruence with what Leary and Tangney (2013) recognized, the participants reported context-congruencies and inconsistencies across different stages of story-based learning (Table 7). To clarify more, the findings associated with Mary’s fluctuations throughout the period reveals that she exceeded the social constraints of the Persian-story-based stage and invested in language learning through the target-story-based activities. Comparing her investment in the source-story and target-story-based activities contributes to understanding some underlying factors regulating language learners’ investment that should not be underestimated. Confirming the results reported by Freire and Branco (2019), there was a growing trend of self-development throughout the investigation due to the impacts of pedagogical and cultural practices on self-identifications and social interactions.

Another remarkable result related to their language investment in the story-based instruction and the future aspiration stages is the participants’ associating self-images with their tendency to enhance symbolic resources. For instance, in the native story-based stage, Fatima highlighted developing her L2 linguistic resources for participation. Arman underlined his propensity to strengthen L1 and L2 linguistic and cultural resources in target story-based participations, and Arshia focused on fostering his linguistic resources in this stage. Moreover, Arman, Azin, and Arshia reported their willingness to enhance both linguistic and cultural resources, but Fatima longed for more intercultural competence in the future (Table 7). The findings can provide evidence for confirming the relationship between learner identity and language investment by Norton (2013) and Sung (2020). As Norton (2013) asserts, L2 learners in different identity positions can partly reframe their access to resources and their relations with the social contexts leading to divergent states of identity reconstruction. Fatima, Arman, and Arshia avoided participating in particular classroom practices despite being highly motivated. Fatima highlighted the sociocultural beliefs regulating her participation in the native-story-based activities. Likewise, Arman and Arshia attributed non-participation in the target-story activities to observing the social structures constraining self-expression (Table 7). Accordingly, the avoidance might be connected to the role of diverse positions and power relations in different social contexts, constraining the possibilities to represent their agency and express their views (Block, 2007; Norton and Toohey, 2011).

The first research question which dealt with the participants’ identity representation in the initial stages of learning English, all participants, even Fatima, embarrassed to enter a different world, highlighted the socialization dimension of language learning. While describing their attitudes toward learning English and its native speakers, no participants prioritized the socioeconomic benefits of learning English in this stage (Table 3).

The first part of the second research question concerns different representations of the participants’ identity construction in the translated Persian story-based instruction stage. They all attributed partaking in most practices to their willingness to communicate their values and views to their classroom community despite tolerating some ambiguities (Table 4). They associated their class participation in this stage with the desire to socialize with their immediate community members, the professor and peers, rather than their imagined communities. In accordance with what Kubota (2011) recorded, they longed for social inclusion into the community of like-minded people. It could be inferred that they attributed this learning experience to the notion of language consumption and pleasure in their immediate classroom community other than the concept of investment for benefits. It seems that Persian stories translated into English could provide a common ground for language learners to express themselves safely owing to their rich cultural background.

Regarding the second part of the second research question related to identity construction in the target-story-based instruction stage, referring to their emotions as an emergent element of identity construction, they all reported the pleasure of understanding intercultural contrasts between the source and target cultures (Table 5). Both Fatima and Arshia attributed their positive feelings in target story-based instruction to the opportunities provided to express personal views and preferences in culturally contrasting situations more easily. The findings also show that despite prioritizing diverse resources, namely cultural and linguistic, all deemed enhancing the resources as a means to the end of socialization within their community. Concerning individual diversities, Fatima was the only participant highlighting the role of target story-based instruction in fostering intercultural relations with target communities. Azin recorded the next varied case of this stage. She experienced an ensuing struggle between target culture learning and native culture attachment resulting in destabilized identities. According to Block (2007), these gaps and contradictions underlie emotional ambivalence as the natural state of uncertainty and discomfort. As Azin pointed out, consuming language to clarify the emerging ambiguity seems to motivate her to engage in language learning activities (Table 7). In other words, her desire to use language for emotional regulation might compel her to engage in activities leading to language learning.

Confirming the previous studies (Kubota, 2011; Chen et al., 2020), the investigation reveals that most participants considered language learning as language consumption for pleasure in different stages of the study, except for the last one. In other words, most of them did not expect personal economic benefits while participating in language learning activities.

The third research question covers various reflections of the participants’ potential identities in their aspirations and expectations. All mentioned that their willingness to be active members of national and international communities intrigues them to reconstruct their identities through participation in the target language practices (Teng, 2019). Unlike the findings revealed in the initial stages, all highlight their desire for language investment within imagined communities, be it national or international, to acquire a high command of English for professional benefits in their future identities (Table 6). While Azin wishes for the potential job opportunity as a multilingual professor teaching abroad, the other cases, Fatima, Arman, and Arshia, aspire for linguistic and intercultural competence to enhance their professional skills within national communities. According to the findings, Fatima longs for becoming a professional teacher, Arman for a Persian writer, and Arshia for a tour guide (Table 7). This corresponds to the findings that although language investment in EFL contexts might contribute to raising socioeconomic status, adult EFL learners do not necessarily long for integrating into the global workplace because of the potential to establish professional opportunities in their home country (Kubota, 2011).

Different categories of language consumption for pleasure include learning a language for socialization, self-esteem, acquiring new and exciting knowledge, and self-actualization in imagined communities (Kubota, 2011). According to the findings related to their future identity, four participants documented language consumption for pleasure in varied forms. Fatima and Arshia desire to use English to socialize with target language members visiting their city. They enjoy making a connection with the tourists. Arshia highlights his aspiration to rapport with them. Moreover, Azin and Arshia use language to convey their national identity and dignity. Arman’s focus is on raising self-esteem through international language consumption.

To conclude, employing theory triangulation and looking at the findings through different lenses of language investment and consumption reveals mixed views about the participants’ aspirations to learn English to enhance their capital or consume the language for pleasure. Various sociocultural factors regulating language learners’ preferences might justify diversities.

### Pedagogical implications of the study

This study can be one of the initial studies on English learners’ aspirations in the EFL context, offering researchers and scholars to broaden their horizons to explore language learners’ identity construction. It is likely a crucial step in understanding how language learners, as language investors or consumers, construct their identities in the community of EFL classrooms. Incorporating both source and target short stories into English language teaching opens up possibilities for new identity reconstructions and representations not necessarily possible in target-story-based instructions. Experiencing diverse identities while learning English might help meet individual differences and varied preferences of language learners. Most importantly, learning English through stories can fulfill the main objective of language use, that is, socialization.

Furthermore, the findings would raise the awareness of EFL curriculum designers and materials writers to plan courses and design materials providing opportunities for learners to experience multiple identities as language investors and consumers. Investigating the complexities of identity development, language investment, and consumption can enhance language teachers’ and learners’ knowledge of challenges and opportunities within language teaching contexts (Norton, 2000).

### Limitations and further suggestions

The initial challenge the researchers confronted was to find students who would participate willingly in this longitudinal research study. While conducting this study, the researcher encountered several limitations; for example, the limited access to the participants for one semester, the accessibility difficulty in the next semester, and conducting a large number of interviews. As mentioned earlier, this study was conducted when the students were in the third semester and they had enrolled in the course entitled ‘Oral Reproduction of short stories.’ The participants were available for a maximum of 4 months when simultaneously they had to attend the compulsory course. But in the following semester, that is, when the participants were in their fourth semester, it became increasingly difficult. Due to the time-consuming nature of the investigation, participants should have had adequate understanding and patience to help in the data collection process through different instruments of questionnaires, interviews, and diaries; this made two participants leave the study in the middle of the process. The researchers had WhatsApp contact with the participants in a convivial atmosphere throughout the data collection procedure.

As Larsen–Freeman (2019) acknowledged, the methodologies implemented to investigate L2 learners’ identity construction are not clear-cut due to its complex and dynamic nature. Some more limitations seem plausible due to the nature of data collected through different self-reporting instruments. Due to the context-congruent nature of qualitative research, the current findings are not generalizable to other contexts (Creswell, 2007). Identity construction requires more longitudinal approaches to theorize the effective identities leading to more productive learning outcomes in the language communities. Applying classroom observations to observe how the participants act and interact (Hennink et al., 2020) in literature classes adds value to prospective studies. Other scholars can study the relatively under-researched foreign language classroom identity. A new avenue for further research can be investigating gender-related aspects of non-native learners’ identity to examine whether their preferences for benefit or pleasure are gender-related.

## Data availability statement

The raw data supporting the conclusions of this article will be made available by the authors, without undue reservation.

## Ethics statement

The studies involving human participants were reviewed and approved by the Department of English Language, Yazd University. The patients/participants provided their written informed consent to participate in this study.

## Author contributions

FS was involved in the data collection procedure. Both authors have analyzed the collected data and contributed to the development of the manuscript.

## Footnotes

1 Materials available upon request from the corresponding author.

## Appendix A

Semi-structured interview

• How can the story affect your interactions and connections?
• What motivated you to participate in classroom interactions?
• How can the class participation affect your self-perception?
• How can you compare your current self-image to your self-image of the beginning stages of learning English?
• How can reading the stories affect your *future* interactions?
• How can reading the stories affect your expectations?

## Appendix B

**APPENDIX TABLE B1:** Some samples of the recurrent codes and themes emerging in different phases of the study.

Participants	Self-descriptions of the initial stages of language learning		Native story-based self- descriptions		Target story-based self-images		Future aspirations	
				
	The most frequent codes	Themes	The most frequent codes Themes	Themes	The most frequent codes Themes	Themes	The most frequent codes	Themes
Fatima	Native speakers were aliens with major cultural differences (Int-1-Oct-22-2019).	Attitudinal aspect	Similarity to our Islamic cultural values motivated me to participate in discussions (Int-2-Nov-5-2019 and Int-6-Dec-10-2019).	Attitudinal aspect	Contrasting my prior perspective, I can adopt cultural diversities more (Int-8-Dec-24-2020 and Ques-2-Dec-3-2019).	Attitudinal aspect	Raising my intercultural competence through studying foreign literature can make me a professional teacher (Dia-9-Jan-21-2020 and Int-9-Jan-21-2020).	Language investment
	I was frustrated and not confident enough about entering a strange world of superiors (Dia-1- Oct-22-2019).	Emotional aspect	I had a mixed feeling of sadness, sympathy, admiration and excitement (Int-3-Nov-12-2019 and Int-6- Dec-10-2019).	Emotional aspect	Participating in class interactions fosters my positive self-image since I can express my emotions and views there (Int-4-Nov-19-2019 and Int-5-Nov-26-2019).	Emotional aspect	I love to transmit my Persian culture and enhance intercultural awareness in return (Dia-9-Jan-21-2020 and Int-9-Jan-21-2020). As a communicative teacher, I would like to have more conversation with the tourists of Yazd (Dia-9-Jan-21-2020 and Int-9-Jan-21-2020).	Consumption for pleasure
			As an active student, I only talk about the events in line with cultural values (Dia-3- Nov-12-2019 and Dia-6-Dec-10-2019).	Social aspect	Self-expressive/more balanced in intercultural relations (Int-8-Dec-24-2020 and Ques-3-Dec-31-2019)	Social aspect		
Arman	Western people seemed attractive and their culture was learnable (Ques-1-Oct-8-2019).	Attitudinal aspect	Taking part in practices encourages me to reconstruct my values leading to the expansion of my identity (Int-3-Nov-12-2019 and Int-6- Dec-10-2019).	Attitudinal aspect	I can strengthen my L1 and L2 cultural and linguistic repertoire through target story- based activities (Dia-8- Dec-24-2020 and Int-8-Dec-24-2020). Sometimes I am reluctant to participate in class activities and learn English (Int-8-Dec-24- 2020).	Attitudinal aspect	I would like to be a well-known writer writing in Persian (Ques-4-Jan-7-2020) This knowledge can lead to a new way to earn a living out of it (Ques-4-Jan-7-2020).	Language investment
	I surprisingly enjoyed the beautiful world of English learning (Dia-1-Oct-22-2019).	Emotional aspect	I admire the current social issues of the stories (Dia-3-Nov-12-2019 and Ques-2-Dec-2019)	Emotional aspect	I like to discuss English stories providing a chance to analyze issues deeply. I should consider cultural constraints (Dia-7-Dec-17-2019 and Int-8-Dec-24- 2020).	Emotional aspect	Besides, I enjoy raising my self-esteem because of having communication skills in international language (Dia-9-Jan-21-2020 and Ques-4-Jan-7-2020)	Consumption for pleasure
			An expressive participant (Dia-6-Dec-10-2019 and Int-3-Nov-12-2019)	Social aspect	An advocate of challenging and contrasting situations (Int-8-Dec-24- 2020 and Ques-3-Dec-31-2019).	Social roles		
Azin	Native speakers were perfect models to learn from (Dia-1-Oct-22-2019).	Attitudinal aspect	Because of my gregarious personality, participating in the practices opens up room for more interactions with my professor and classmates (Dia-2- Nov-5-2019 and Int-2-Nov-5-2019)	Attitudinal aspect	Clarifying the ambiguity and regulating my emotions compel me to engage in discussions and learn the language (Int-4-Nov-26-2019).	Attitudinal aspect	I intend to be a resourceful bilingual with rich linguistic and cultural competence (Int-9-Jan-21-2020 and Ques-4-Jan-7-2020)	Language for investment
	I was not really satisfied because I did not understand their culture (Int-1-Oct-22-2019).	Emotional aspect	I am an eager analyzer of the story even when I am not positive (Int-6-Dec-10-2019 and Ques-3-Dec-31-2019).	Emotional aspect	Experiencing the puzzling feelings of acceptance or rejection of conceptualizations in target and source cultures is a motivating factor (Int-4-Dec-24-2020).	Emotional aspect Social roles	I hope to be a multilingual professor teaching language and literature at a foreign university (Dia-9-Jan-21-2020 and Int-9-Jan-21-2020)	
			An eager analyzer (Int-2-Nov-5-2019 and Int-6-Dec-10-2019 and Ques-3-Dec-31-2019)	Social aspect	Ambiguity clarifying learner (Int-5-Nov-26-2019 and Ques-2-Dec-3-2019).	Social aspect	I love to convey the ignored heritage of Persia to foreigners (Int-9-Jan-21-2020)	Language consumption for pleasure
Arshia	I thought that English people were superior and I wanted to forget Persian (Int-1-Oct-22-2019).	Attitudinal aspect	To me, language learning in this context means self-confidence, self-expression, and self-development (Dia-6-Dec-10-2019 and Int-2-Nov-5-2019).	Attitudinal aspect	To raise my self-confidence associated with my proficiency level, I always participated actively (Dia-7-Dec-17-2020 and Int-7-Dec-17-2020).	Attitudinal aspect	I am going to be a successful multicultural tour guide (Int-9-Jan-21-2020 and Ques-4-Jan-7-2020)	Language for investment
	I enjoyed learning English abroad because of being somehow easy and pleasant (Qtn-1-Oct-8-2019).		I appreciate Persian history and culture (Dia-2- Nov-5-2019 and Int-2-Nov-5-2019).		Challenging situations inspired me to vocalize my personal preferences (Dia-7-Dec-17-2020 and Int-7-Dec-17-2020).		While communicating with them, I am pleased to express my national dignity effectively (Int-9-Jan-21-2020 and Ques-4-Jan-7-2020)	Language consumption for pleasure
			A communicative learner (Int-2-Nov-5-2019 and Ques-2-Dec-3-2019)		An always active participant (Dia-7-Dec-17-2020 and Int-4-Nov-19-2019 and Int-7-Dec-17-2020).		When my proficiency level is high, I can afford to make rapport with them easier and faster (Dia-9-Jan-21-2020 and Ques-4-Jan-7-2020)	
